# Inoculum Sources Modulate Mycorrhizal Inoculation Effect on *Tamarix* *articulata* Development and Its Associated Rhizosphere Microbiota

**DOI:** 10.3390/plants10122716

**Published:** 2021-12-10

**Authors:** Karima Bencherif, Frédéric Laruelle, Yolande Dalpé, Anissa Lounès-Hadj Sahraoui

**Affiliations:** 1 Départements des Sciences Agrovétérinaires, Faculté des Sciences de la Nature et de la Vie, Université de Djelfa, Route de Moudjbara, Boite Postale 3117, Djelfa 17000, Algeria; 2 Unité de Chimie Environnementale et Interactions sur le Vivant (UCEIV), Université du Littoral Côte d’Opale, UR 4492, SFR Condorcet FR CNRS 3417, 50 rue Ferdinand Buisson, CEDEX, 62228 Calais, France; frederic.laruelle@univ-littoral.fr (F.L.); anissa.lounes@univ-littoral.fr (A.L.-H.S.); 3 Centre de Recherche et Développement d’Ottawa, Agriculture et Agroalimentaire Canada, 960 Carling Ave., Ottawa, ON KIA 0C6, Canada; yodalpe@gmail.com

**Keywords:** mycorrhizal inoculation, phospholipid fatty acids, ergosterol, soil salinity

## Abstract

(1) Background: Soil degradation is an increasingly important problem in many parts of the world, particularly in arid and semiarid areas. Arbuscular mycorrhizal fungi (AMF) isolated from arid soils are recognized to be better adapted to these edaphoclimatic conditions than exogenous ones. Nevertheless, little is known about the importance of AMF inoculum sources on *Tamarix articulata* development in natural saline soils. Therefore, the current study aims at investigating the efficiency of two AMF-mixed inoculums on *T. articulata* growth, with consideration of its rhizosphere microbiota. (2) Methods: indigenous inoculum made of strains originating from saline soils and a commercial one were used to inoculate *T. articulata* in four saline soils with different salinity levels under microcosm conditions with evaluation of rhizosphere microbial biomasses. (3) Results: Our findings showed that indigenous inoculum outperforms the commercial one by 80% for the mycorrhizal rate and 40% for plant biomasses, which are correlated with increasing shoot phosphorus content. Soil microbial biomasses increased significantly with indigenous mycorrhizal inoculum in the most saline soil with 46% for AMF, 25% for saprotrophic fungi and 15% for bacterial biomasses. (4) Conclusion: Present results open the way towards the preferential use of mycorrhizal inoculum, based on native AMF, to perform revegetation and to restore the saline soil microbiota.

## 1. Introduction

Mediterranean ecosystems are severely subjected to soil desertification [1,2], which negatively affects plants establishment, growth and biodiversity as well as multiplicity and richness of rhizospheric microbiota [3,4,5]. In Algerian arid and semi-arid areas, soil desertification due to different abiotic stress, especially soil salinity, restricted natural process of plant succession [1,6,7]. Furthermore, salinity is recognized as a severe global ecological problem being one of the most influential abiotic factors limiting plant growth and yield in arid and semi-arid areas. For purposes of definition, saline soils are those which have an electrical conductivity of the saturation soil extract of more than 4 dS·m^−1^ at 25 °C [8]. To counteract different problems of soil degradation, the Algerian state put in place a revegetation program based on indigenous shrubs [2,9]. However, these programs were relatively unproductive due to conventional planting techniques based on cutting methods not adapted to local stressed environmental conditions [10]. In fact, steppic arid and semi-arid Algerian areas are subject to several types of abiotic stresses, such as salinity, drought, calcareous, etc., which deteriorate their soils and makes them inappropriate for cultivation [2,9,10].

*Tamarix articulata* Vahll was one of the proposed halophytic plants in the revegetation programs. It has interesting ecological and medicinal values such as erosion control, windbreak, and applications in apiculture, cosmology, leather and timber industries [11,12]. *T. articulata* is also known for its osmoregulation potential in saline environments [13]. Nevertheless, *T. articulata* plantation in steppic areas was performed using classical practices, which led to the death of more than 70% of the total plantation [9]. In addition, excessive steppic soil salinization negatively affects seed propagation of *T. articulata* and limits plant growth and rhizosphere microbiota diversity [3,4,5,6,7,8,9,10]. Salinity also decreases phosphorus concentrations in *Tamarix* tissue since phosphate ions precipitate with Ca^2+^, Mg^2+^ and Zn^2+^ ions and become unavailable [5].

Arbuscular mycorrhizal fungi (AMF) bio-fertilization is a recognized and valuable strategy to restore disturbed areas [1,14,15]. It is recognized that AMF, which belongs to the Glomeromycota phylum [16], are associated to plant root systems in most natural and agro-systems including stressed soils [5,17]. In addition, their benefits to plants growing in saline soils have been demonstrated [17,18,19,20]. Several studies have suggested that inoculation with AMF isolated from saline soils can alleviate salt stress more efficiently than those originating from non-saline soils [5,6,15,18,20]. For example, Garg and Panday (2016) [14] demonstrated the effectiveness of indigenous AMF ecotype, *Acaulospora*, *Glomus* and *Gigaspora* genera, isolated from saline soils as compared to introduced ones (*Funneliformis mosseae* and *Rhizophagus irregularis*) under different soil salinity levels.

Thus, the use of AMF for *T. articulata* plantations could constitute a good strategy to overcome the negative impact of abiotic saline stresses and increase plant productivity under these harsh conditions. However, it is imperative to select the best AMF strains-*T. articulata* combination. Previous study established by Bencherif et al. (2015) [21] in the four steppic degraded localities; Laghouat (LG), Hassi Bahbah (HB), Djelfa (DU) and Boughzoul (BG) indicated that *T. articulata* is a mycotrophic plant. However, little is known about the impact of AMF inoculum sources on its growth in natural arid and semi-arid saline soils. For that, the objective of the present study is to investigate whether the indigenous vs. commercial AMF inoculum can influence the growth of *T. articulata* and their associated microbial communities. For this purpose, soil samples selected from the four saline localities were re-inoculated by two different inoculum: a commercial inoculum (Symbivit, Inoculum Plus, Bretenière, France) as compared to an indigenous one containing AMF strains isolated from the saline soils, for a period of 6 months. We hypothesize that natural soil salinization ranging from 1.1 to 4.5 dS·m^–1^ will reduce establishment of AMF symbiosis and negatively affect rhizospheric microbiota, leading to a decrease of mycorrhiza effects on plant parameters. Secondly, as soil salinization modifies microbiota status, an additional hypothesis is to determine the effect of AMF inoculum source on rhizosphere microbiota biomasses. Finally, our third hypothesis is that the efficiency of inoculation depends on AMF inoculum source owing to different phenotypic characters.

## 2. Results

### 2.1. AMF T. articulata Colonization and Diversity

#### 2.1.1. AMF *T. articulata* Colonization

In order to evaluate the impact of AMF inoculum source on *T. articulata* colonization and diversity, the mycorrhizal rate was evaluated after 6 months of culture on non- saline soil Laghouat (LG), two slightly saline soils Hassi-Bahbah (HB), Djelfa (DU) and on the moderate saline soil Boughzoul (BG). The highest mycorrhizal colonization rate for the non-inoculated (NI) plants was recorded in non-saline soil LG (15.4%), with a decrease of 60% in the moderate saline soil BG. It is worth noting that the highest mycorrhizal colonization rate was observed with indigenous inoculum (AI) in moderate saline soil BG (33%). In addition, the mycorrhizal rate with indigenous inoculum was about 65, 61, 51 and 45% higher than with non-inoculated soils in LG, HB, DU and BG soils, respectively (Table 1). 

In the presence of commercial inoculum (CI), mycorrhizal colonization rates were 37, 38 and 48% lower than in non-inoculated soils in LG, HB and DU soils, respectively. Interestingly, mycorrhizal colonization increased with commercial inoculum in moderate saline soil BG by 16% compared to that in non-inoculated soil. Nevertheless, indigenous inoculum increased mycorrhizal colonization by approximately 69%, 61%, 80% and 80% when compared with a commercial one, respectively, in the four studied soils. In order to evaluate the impact of mycorrhizal colonization on the different measured parameters under the inoculation treatments, Pearson’s correlation coefficients were evaluated (Table 2). 

That analysis indicated a positive impact on soil microbial biomasses under indigenous inoculation treatment and was recorded by positive correlation between the mycorrhizal colonization rate, AMF biodiversity (*p* < 0.01), the ergosterol amount (*p* < 0.05), the sum of PLFA for Gram negative bacteria and with Gram positive bacteria (*p* < 0.05). One-way ANOVA revealed a negative influence of soil salinity on the mycorrhizal colonization rate (*p* < 0.04), with a positive influence of indigenous inoculum on mycorrhizal rates at *p* < 0.001 (Table 3).

#### 2.1.2. AMF Spore Diversity

After six months of culture, AMF spore abundance in 100 g of each studied saline soil collected from *Tamarix* pot cultures was determined (Supplementary Materials; Table S1). Total spore numbers were higher with indigenous AMF inoculum in all studied saline soils as compared with the commercial one with an abundance of: *Funneliformis geosporum*, *F. mosseae* and *Septoglomus constrictum*. The AMF species’ richness and diversity (Shannon index) increased significantly with indigenous AMF inoculation treatment. Detrended Correspondences Analysis (DCA) showed that AMF spore species are associated with indigenous inoculum in the four studied soils (Figure S1). Positive correlation was recorded between the mycorrhizal colonization rate, the AMF species richness (r = 0.94 **, *p* < 0.01) and biodiversity indices under indigenous inoculation treatment (Table 2). However, non-significant correlation was recorded between the AMF species’ richness and the mycorrhizal colonization rate under commercial inoculation treatment (r= −0.4, *p* < 0.2; Table 2). The positive impact of indigenous inoculum on AMF biodiversity was recorded by the ANOVA test at *p* < 0.001 (Table 3).

### 2.2. Effect of Mycorrhizal Inoculation on T. articulata Growth

#### 2.2.1. Seedling Biomasses

**Figure 1:**
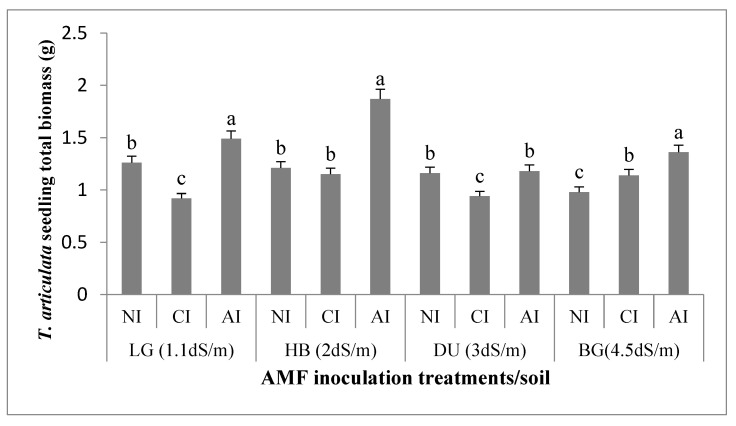
Effect of AMF inoculum sources on *T. articulata* total seedling biomass in the four studied soils. NI: non-inoculated. CI: commercial inoculum. AI: Indigenous inoculum. Data are represented as mean ± standard deviation. Means are obtained from five replicates (*n* = 5). Different letters indicate significant differences between treatments in the four studied soils according to the Tukey HSD test (*p* < 0.05).

Soil salinity negatively affects root and shoot biomasses. Root and shoot biomasses decreased by 15% from non-saline soil LG to moderate saline soil BG in non-inoculated treatment (Figure 1). Significant increases of total plant biomasses were observed under indigenous inoculation treatment in LG, HB, and BG soils as compared to non-inoculated treatments (r = 0.86, *p* < 0.001) (Table 2). The increase was respectively approximately 23, 66, and 38% (Figure 1). The total biomass increases was non-significant in DU soil (2%). In addition, significant increase of total biomasses was recorded with indigenous inoculum as compared with commercial one. It was approximately 34, 72, 24 and 38% higher in LG, HB, DU and BG soils, respectively. Positive correlation was recorded between shoot dry weight and different studied parameters under indigenous inoculation, while non-significant correlation was observed with commercial inoculum (Table 2). ANOVA revealed a significant effect of indigenous inoculation treatment on total dry weights (R^2^ = 0.61, *p* < 0.001) (Table 3).

#### 2.2.2. Phosphorus Contents in Soil and Shoots

**Figure 2:**
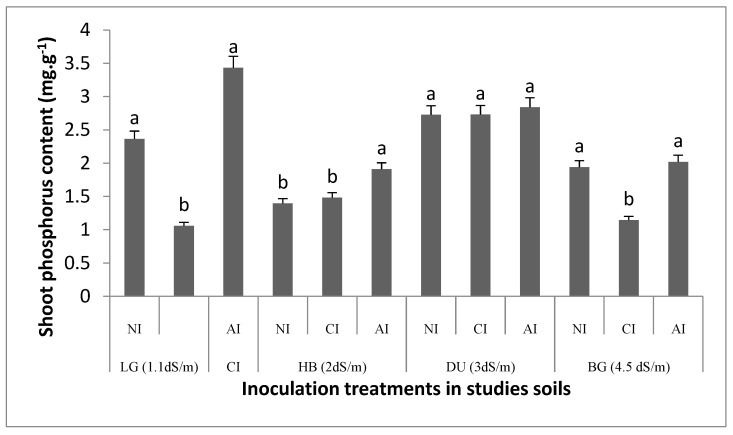
Impact of AMF inoculum sources on *T. articulata* shoots phosphorus content in the four studied soils. NI: non-inoculated. CI: commercial inoculum. AI: Indigenous inoculum. Data are represented as mean ± standard deviation. Means were obtained from five replicates. Different letters indicate significant differences between treatments according to the Tukey HSD test (*p* < 0.05).

In non-inoculated soils shoot phosphorus contents were about 2.3 and 1.94 mg·g^−1^ in (LG) and (BG) soils, respectively. A significant increase of shoot phosphorus content was recorded with indigenous inoculum (Figure 2). These increases were approximately 15 and 10% in LG and BG soils in comparison with non-inoculated plants, and approximately 33 and 17% respectively in the same soils as compared with commercial inoculum. The mycorrhizal colonization rate was positively correlated with shoot phosphorus content under indigenous inoculum (*p* < 0.01) (Table 2). Analysis of variance indicated that shoot phosphorus content was affected positively by indigenous AMF inoculation (R^2^ = 4.34, *p* < 0.05), while no significant effect of commercial inoculation was recorded (R^2^ =1.9, *p* < 0.8) (Table 3).

### 2.3. Influence of Mycorrhizal Inoculation on Soil Microbial Biomass

**Figure 3:**
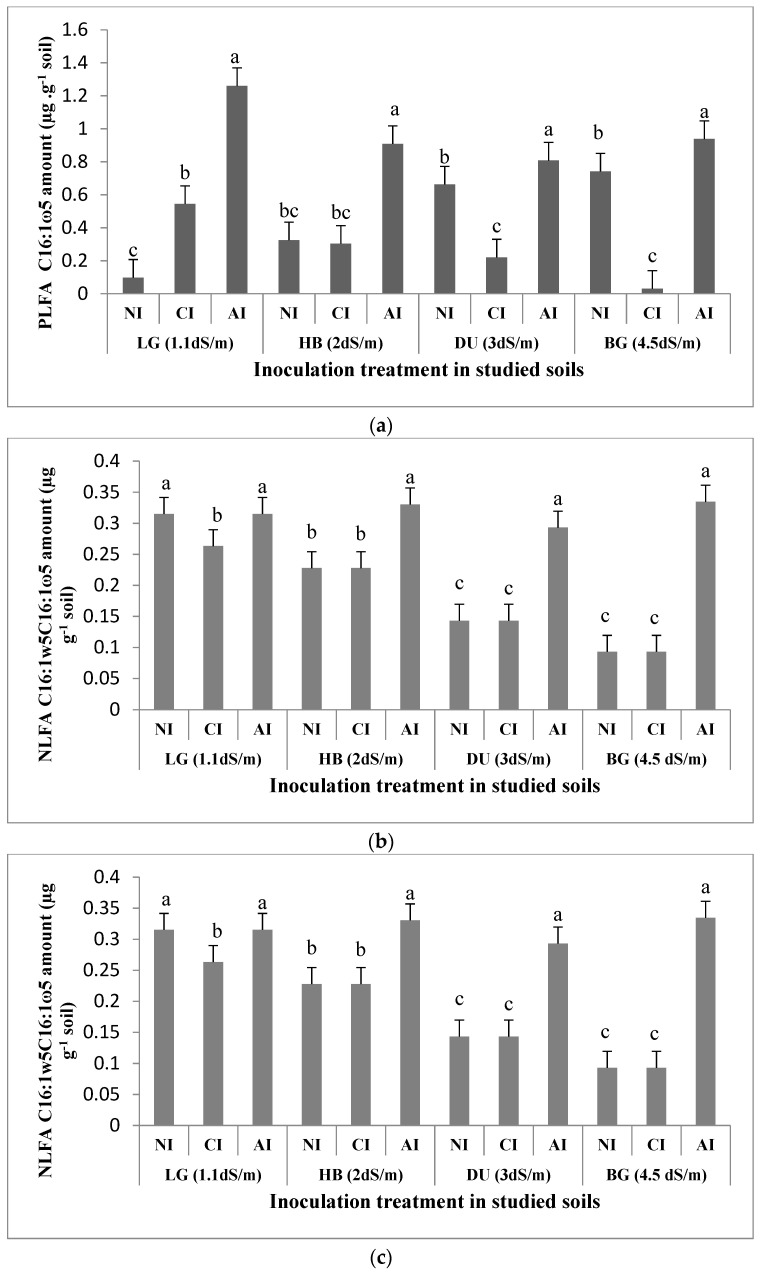
Effect of AMF inoculum sources on C16:1ω5 soil content of *T. articulata* cultivated soils. (**a**) PLFA amount. (**b**) NLFA amount. (**c**) NLFA: PLFA C16:1w5 ratio. NI: non-inoculated. CI: commercial inoculum. AI: Indigenous inoculum. Data are represented as mean ± standard deviation. Means were obtained from five replicates. Different letters indicate significant differences between treatment and different symbols indicate significant differences between soils according to the Tukey HSD test (*p* < 0.05).

In order to determine the impact of AMF inoculum sources on *T. articulata* rhizosphere microbial biomass, specific lipid biomarkers were assessed and compared between the different soils. The amounts of phospholipid fatty acid (PLFA) C16:1ω5, used to quantify AMF in soils, increased with soil salinity level in non-inoculated soils. The amounts ranged from 0.09 µg·g^−1^ in non-saline soil LG to 0.7 µg·g^−1^ in moderate saline soil BG. In the presence of AMF indigenous inoculum, PLFA C16:1ω5 was found to be 12, 58, 21 and 20% higher, respectively, in non-saline soil LG, slightly saline soils HB and DU and in moderate saline soil BG as compared with non-inoculated soils (Figure 3A). In the presence of the commercial inoculum, PLFA C16:1ω5 contents were approximately 71, 60, 58 and 90% lower in comparison with indigenous inoculum in LG, HB, DU and BG soils, correspondingly. While positive correlation was recorded between the mycorrhizal colonization rate and PLFA C16:1ω5 (r = 0.58 *, *p* < 0.02) in the presence of indigenous inoculum, no significant correlation was observed in the presence of commercial inoculum and in non-inoculated soils (Table 2). Significant positive effect of AMF indigenous inoculum on PLFA C16:1ω5 was recorded (*p* < 0.05) while no effect of commercial inoculation was observed (*p* < 0.12). Contrast between indigenous and commercial inoculation treatments revealed a significant difference at *p* < 0.04 (Table 3).

The neutral lipid fatty acids (NLFA) C16:1ω5 soil content, mainly representing AMF vesicle storage structures, were found to be negatively affected by soil salinity level in non-inoculated treatments (Figure 3B) and varied from 0.3 µg·g^−1^ in non-saline soil LG to 0.09 µg·g^−1^ in moderate saline soil BG. Furthermore, inoculum sources significantly affected NLFA C16:1ω5. The highest amount was recorded under indigenous inoculum in the four studied soils independently from the soil salinity level (Figure 3B). They were approximately 11, 15 and 24% higher, respectively, in HB, DU and BG saline soils as compared to non-inoculated soils, with non-significant difference in non-saline soil LG. No significant difference in the NLFA C16:1ω5 amount was recorded between commercial inoculum and non-inoculated treatments except for low saline soil LG where it was about 11% lower with commercial inoculum; the NLFA C16:1ω5 amount was lower with commercial inoculum in comparison with indigenous one. They were approximately 5, 11, 15 and 25% lower, respectively, in LG, HB, DU and BG soils (Figure 3B). Whereas positive correlation was recorded between the mycorrhizal colonization rate and NLFA C16:1ω5 under indigenous inoculation treatment (*p* < 0.02), negative correlation was shown with commercial inoculum (*p* < 0.05) (Table 2). In addition, NLFA C16:1ω5 was positively affected by AMF indigenous inoculation at *p* < 0.002 with a difference between AMF indigenous inoculum and the commercial one at *p* < 0.01 (Table 3).

The NLFA/PLFA C16:1ω5 ratio was found to be much higher in the non-saline soil LG (ratio = 3) than in moderate saline soil BG (ratio = 0.3) in non-inoculated soils, with a positive impact of indigenous inoculum in the four studied saline soils (Figure 3C).

**Figure 4:**
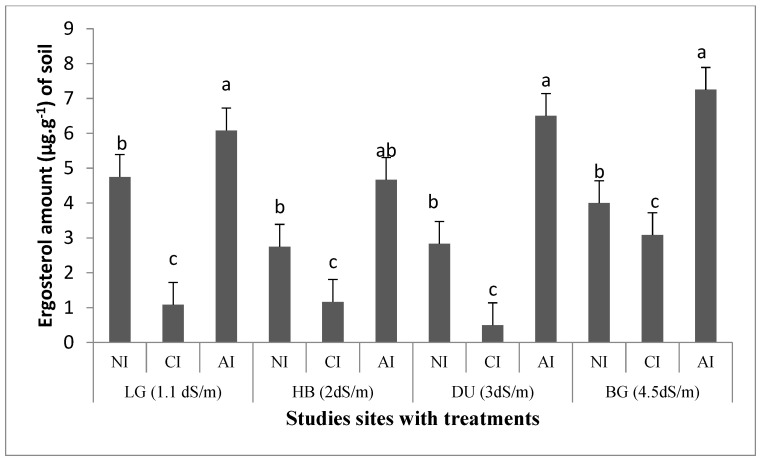
Effect of AMF inoculum sources on ergosterol levels of the soils of *T. articulata* in the four studied soils. NI: non-inoculated. CI: commercial inoculum. AI: Indigenous inoculum. Data are represented as mean ± standard deviation. Means were obtained from five replicates. Different letters indicate significant differences between treatments and different symbols indicate significant differences between soils according to the Tukey HSD test (*p* < 0.05).

Ergosterol content, an indicator of saprotrophic fungi, reached 4.8 µg·g^−1^ in non-saline soil LG, 2.8 µg·g^−1^ in the two slightly saline soils (HB and DU), and 4 µg·g^−1^ in the moderate saline soil BG, than in non-inoculated soils. However, soil ergosterol content was positively stimulated by indigenous mycorrhizal inoculum in all studied soils (Figure 4). It increased by 13, 17, 23 and 19% in LG, HB, DU and BG soils, respectively. In addition, in the presence of commercial inoculum, ergosterol content decreased significantly as compared to non-inoculated soils by approximately 13% and 32% in LG and BG soils, respectively. With indigenous AMF inoculum, the ergosterol amount increased by approximately 75 and 50%, respectively, in BG and LG soils as compared with commercial inoculum.

**Figure 5:**
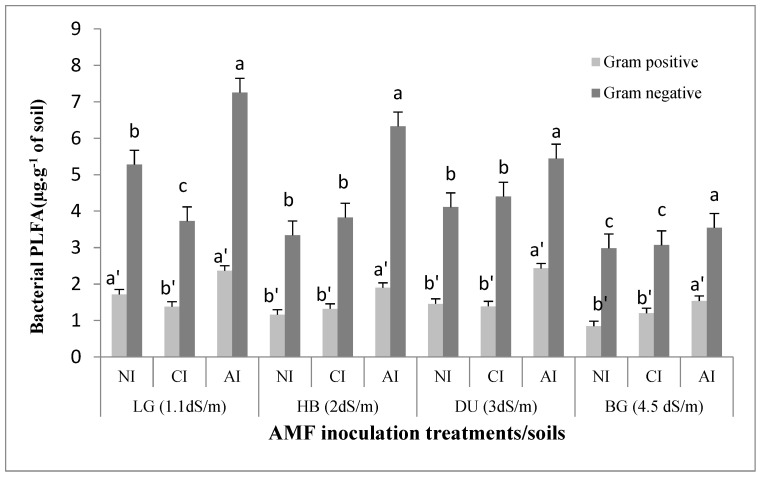
Influence of AMF inoculum sources on PLFA bacteria Gram + and Gram - amounts of the soils of *T. articulata* in the four studied soils. NI: non-inoculated. CI: commercial inoculum. AI: indigenous inoculum. Data are represented as mean ± standard deviation. Means were obtained from five replicates (*n* = 5). Different letters indicate significant differences between treatment and different symbols indicate significant differences between soils according to the Tukey HSD test (*p* < 0.05).

Gram negative bacterial biomass decreased from 5 to 2 µg·g^−1^ between non-saline soils LG and moderate saline soil BG in non-inoculated soils. Similarly, Gram positive bacteria were reduced from 1.7 to 0.8 µg·g^−1^ between LG and BG soils (Figure 5). In the presence of indigenous inoculum, Gram-negative bacterial biomass was significantly increased by 21, 30, 13 and 60% in LG, HB, DU and in BG soils respectively as compared to non-inoculated soils (Figure 5). Positive correlation was recorded between PLFA biomarkers content of Gram negative bacteria and the mycorrhizal colonization rate under indigenous inoculum (r = 0.55, *p* < 0.01) (Table 2). A significant effect of indigenous mycorrhizal inoculation was also noted (*p* < 0.02) (Table 3). Likewise, in the presence of indigenous inoculum, Gram positive bacteria increased by 14, 97, 74 and 69% in LG, HB, DU and BG soils, respectively, compared to non-inoculated soils (Figure 5). Positive correlation was recorded between mycorrhizal colonization rate and PLFA Gram positive bacteria under indigenous inoculum (r = 0.5, *p* < 0.02) (Table 2). The ANOVA test showed positive influence of AMF indigenous treatment on PLFA content of soil Gram positive bacterial biomasses (*p <* 0.01) (Table 3).

## 3. Discussion

The present study aims at evaluating the importance of AMF inoculum sources on *Tamarix articulata* development and its associated rhizosphere microbial biomasses in saline soils.

The current findings clearly show a significant effect of mycorrhizal inoculation and inoculum sources on *T. articulata* shoot and root biomasses, with a positive Pearson’s correlation recorded with indigenous inoculum and non-significant correlation with commercial inoculum under saline conditions. Similar results were reported in previous studies with other plant species (*Cajanus cajan*, *Prosopis juliflora*, *Ocimum basilicum*) under saline conditions [6,15,22]. It seems that under saline stress, indigenous AMF species are more efficient than those contained in the commercial inoculum. The higher performance of AMF strains contained in the indigenous mixed inoculum may be attributed to their better adaptation to saline environmental conditions [5,6,23,24].

It is evident that the establishment of mycorrhizal symbioses to AMF inoculum clearly promotes salinity tolerance through various mechanisms, such as nutrient acquisition, production of plant growth hormones, accumulation of osmoregulatory compounds and increased photosynthetic activity [20,24]. This increased tolerance of *T. articulata* is reflected in its biomass improvement and seemed to be related to phosphorus uptake, which is revealed by the positive exchange observed between the roots and the aerial parts, similar to observations found in previous studies of Evelin et al. (2009) [3], Alqarawi et al. (2014) [14], Fall et al. (2017) [22] and Saxena et al. (2017) [25]. In addition, positive correlation was observed between shoot phosphorus content and shoot dry weight, and also between shoot phosphorus content and mycorrhizal colonization rates under indigenous inoculation treatment. Those correlations suggest that the indigenous AMF inoculum provided a very effective pathway by which phosphorus was scavenged from saline soil and rapidly delivered to *T. articulata* cortical cells within the root, bypassing direct uptake and reducing the impact of Pi depletion in the rhizosphere [25,26]. Similar contribution of AMF symbiosis in phosphorus nutrition enhancement under saline conditions has already been described for several plants such as *Ocimum basilicum* [6], *Cajanus cajan* [15], *Prosopis juliflora* [22], *Olea europea* [23] and *Gossypium hirsutum* [27], but never for *T. articulata*. Smith et al. (2011) [26] reported that the phosphorus amount required by most plants is about 0.2% of plant dry weight. In the present study, shoot phosphorus recorded on the *T. articulta* shoot with indigenous inoculum was greater than 0.2%, while it remained lower than 0.2% with the commercial inoculum. This is in accordance with several previous studies which highlighted the efficiency of indigenous AMF inoculation to improve the uptake of available phosphorus under saline conditions [6,15,22].

Moreover, commercial and indigenous inoculums both have the same two species (*F. mosseae* and *F. geosporum*) but the efficiency of AMF indigenous inoculum suggested that they are different strains. Garg and Penday (2016) [15] explained that AMF strains used in commercial inoculum are generalist and perform under a variety of conditions, but are not necessarily the best overall. That was proved by the results of present study when the indigenous strain had better performance than the commercial one on *T. articulata* plant development and on microbial rhizosheric biomasses under studied saline conditions.

The enhancement of *T. articulata* biomass with indigenous AMF inoculum might be not only attributed to a better mycorrhizal colonization rate, but also to the stimulation of associated rhizosphere microbiota. Indeed, a positive correlation was recorded between the mycorrhizal colonization rate and AMF biodiversity indices under indigenous inoculation treatment. Increases of AMF species’ richness and the Shannon biodiversity index with indigenous mycorrhizal inoculum in comparison with the commercial one was previously reported on olive tree and in sweet basil cultivated under saline soil conditions [6,23]. In addition, our results indicated that indigenous inoculum stimulated total soil microbial biomasses after 6 months of culture. A positive correlation between the mycorrhizal colonization rate and specific lipid biomarker contents (NLFA C16:1ω5, PLFA C16:1ω5, total PLFA specific for Gram positive and Gram negative bacteria) was recorded. A positive impact of indigenous AMF inoculum was clearly observed on total bacterial and fungal biomasses in the different studied soil salinity levels. This is in accordance with Mechri et al. (2014) [28]; their research reported a significant effect of mycorrhizal inoculation on microbial biomass in olive trees (*Olea europea*) under saline conditions.

The positive impact of indigenous AMF inoculum on soil microflora biomasses may depend on the deployment in the rhizosphere of an extensive network of extra-radical AMF mycelium which, while degrading, fed soil microorganisms with an additional source of organic matter and consequently influenced soil chemical composition and contributed to microflora enrichment [25,29]. The positive effect of indigenous inoculum on rhizospheric microbial biomasses may also be attributed to the ability of the indigenous AMF strain to preserve a stable K^+^/Na^+^ ratio [30]. This helps the rhizospheric bacterial and fungal communities to maintain their enzymatic process stable under saline stress conditions [20,29,31]. The lower mycorrhizal potential of AMF commercial inoculation slowed down the development of the soil microbial community, and delayed the development of saprotrophic fungi and bacterial communities in the studied saline soils.

## 4. Materials and Methods

### 4.1. Experimental Sites

Soil samples were collected from the rhizosphere of *T. articulata* shrubs growing in four experimental sites located in degraded arid and semi-arid Algerian steppic areas. They were classified according to their soil salinity levels (One non- saline soil Laghouat (LG) 1.1 dS·m^–1^(32°55′ N, 2°30′ E), two slightly saline soils Hassi Bahbah (HB) 2 dS·m^–1^ (35°04′ N, 3°13′ E), Djelfa (DU) 3 dS·m^–1^ (34°40′ N, 3°15′ E) and one moderate saline soil Boughzoul (BG) 4.5 dS·m^–1^(35°42′ N, 2°50′ E)). The studied sites are geographically separated by about 100 km between LG and DU, 50 km between DU and HB, and 75 km separates the HB and BG sites. The four studied soils are characterized by their low available phosphorus, which ranged between 0.1 mg·Kg^−1^ (DU) and 0.2 mg·Kg^−1^ (BG). Soil pH was between 7.4 and 8. These soils were chosen for their degradation due to the salinity effect and for the low efficiency of revegetation programs established in these localities. Physico-chemical characteristics of the soils are presented in Supplementary Materials (Table S2). All details of the studied sites (location and soil characteristics) were described in Bencherif et al. (2015) [21].

### 4.2. Treatments and Experimental Design

Cuttings of *T. articulata* shrubs were stratified at 4 °C for 15 days and transplanted in pot culture (300 mL) filled with a substrate made of 50% unsterilized studied site soils and 50% with sterilized sand (autoclaved at 120° for 2 h) (*V*:*V*, 1:1). After the substrate mixture preparation (50% natural soil + 50% sterilized sand), the salinity level was measured (LG soils 1.1 dS·m^−1^, HB soil with 2 dS·m^−1^, DU soil with 3 dS·m^−1^, and BG 4.5 dS·m^−1^). The experimental design was full factorial in randomized block. Two types of AMF inoculum were used in this study: A commercial (Inoculum Plus: Symbivit, Bretenière, France) mix (CI) consisting of six Glomeromycotina species (*Rhizophagus irregularis* BEG140, *Funneliformis mosseae* BEG95, *Claroideoglomus etunicatum* BEG92, *Claroideoglomus claroideum* BEG96, *R. microaggregatum* BEG56 and *F. geosporum* BEG199) in an inert substrate, and an indigenous inoculum (AI) containing eight Glomeromycotina species (*Septoglomus constrictum*, *Funneliformis geosporum*, *F. mosseae*, *F. coronatum*, *F. caledonium*, *Oehlia diaphana*, *R. fasciculatum* and *Gigaspora gigantea*). This indigenous inoculum was obtained in an open pot culture of *Medicago sativa* containing natural saline soils with 4.5 dS·m^–1^. Indigenous inoculum was made of a mixture of AMF spores, mycorrhizal roots plus soil containing mycelium. For each treatment 500 propagules were added in the pot cultures. Non-inoculated (NI) treatments received the same amount of autoclaved inoculum (autoclaved at 121°C for 30 min over two consecutive days) (Changey et al., 2019) [32]. Five replicates were performed for each treatment.

Pots were grown for six months under day/night 16/8 h home periods, 25/22 °C, 70% relative humidity, and light intensity 110 µE/m^2^·s). After the growth period, plants were uprooted; root systems were gently washed under water, and shoots and roots were dried (72°C for three days). After drying, plant tissue biomasses were weighed, then ground, hashed, digested in 2 mL HCl (1N) and 10 mL HNO_3_ and then analyzed by the colorimetric method for phosphorus [33]. Using the same colorimetric method elaborated by John (1970) [33], soil phosphorus was analyzed in all studied cultures.

### 4.3. Soil Microbial Analysis

#### 4.3.1. Description of AMF Communities

After 6 months of culture, 100 g of soils from each pot culture were sampled for AMF spore isolation using the wet sieving method [34]. Spores were counted under stereomicroscope and grouped according to morphological characteristics. Spore abundance was evaluated by counting the number of spores per identified or characterized species. Blaskowski (2012) [35], INVAM and the Blaszkowski website were used. Roots were cleared and stained according to the Phillips and Hayman [36] method was modified for microwave ovens by Dalpé and Séguin (2013) [36]. Roots were washed under water to remove soil debris and cut into 10−15 mm fragments to allow better staining performance. *Tamarix* roots required 5 min H_2_O_2_ bath to remove pigments. A microwave modified staining procedure was used to bleach and stain roots [36,37]. Roots were bleached in KOH 5%, 25 s in a microwave oven (800 W), rinsed three times in water and acidified for 5 min in HCl 1 N. Roots were stained in Trypan blue (5%) in lactic acid glycerol water (1:1:1, *V*:*V*:*V*) for 45 s in a microwave oven (800 W). The excess staining was removed in a bath of glycerol-water solution (1:1, *V*:*V*). The mycorrhizal, arbuscular and vesicle rates were estimated using the magnified gridline intersect method [38]. Two thousand seven hundred root fragments from the 60 cultured seedlings were observed under an optical microscope (×100); intersections were counted in the following categories: negative (non-fungal material in root), arbuscular, vesicles and hyphae.

#### 4.3.2. Fatty Acid Analysis

Microbial biomass was determined on soil pot culture after 6 months of culture by the use of the fatty acid methyl ester (FAME) method [39]. The amounts of the phospholipid fatty acids (PLFA) C16:1ω5 and the neutral lipid fatty acids (NLFA) C16:1ω5 were determined and used as indicators of AMF biomass [39]. The NLFA/PLFA ratio of C16:1ω5 fatty acid was calculated; when it was superior to 1, it indicated that the C16:1ω5 fatty acid was originating from AMF and not from bacteria. Gram positive bacteria biomasses were quantified by the sum of the PLFA: i15:0, a15:0, i16:0, i17:0, a17:0, and Gram negative bacteria biomass quantified by the sum of the PLFA: cy17:0, C18:1ω7 and cy19:0 amounts [40].

#### 4.3.3. Ergosterol Extraction

The soil biomass of saprotrophic fungi was quantified by analyzing free ergosterol content using 4 g of freeze-dried soil from each studied site. Ergosterol was extracted in 5 mL of methanol in the dark. The samples were mixed in a vortex apparatus for 1 min, extracted overnight and then refluxed at 70 °C for 90 min. After cooling, 1 mL H_2_O and 2 mL cyclohexane were added. The samples were mixed in a vortex apparatus for 20 s, centrifuged for 5 min at 3000 rpm. After removal of the upper phase, ergosterol was extracted from the methanol fraction with further 1.5 mL cyclohexane with evaporation under N_2_. Final extracts were analyzed with the use of GCMS [21].

### 4.4. Statistical Analysis

Data were analyzed statistically by means of comparison by multivariate analysis with Excel stat 2020, using the Tukey test (HSD b 5%). A two-tailed Pearson correlation test was carried out between the mycorrhizal colonization rate and the shoot dry weight as independent quantitative variables and different measured parameters and as dependent quantitative variables, which were: AMF species richness, Shannon Biodiversity index, shoot and root dry weight, shoot and soil phosphorus content, shoot protein content, Gram positive and Gram negative bacteria, PLFA and NLFA C16:1ω5, as well as ergosterol content. All measurements were performed five times for each treatment, and the calculated means and standard deviation were mentioned. One-way ANOVA with defining specific contrast for single factor (salinity/inoculum source) with three levels was performed on root mycorrhizal rate, phosphorus content, total biomasses and biodiversity indices using Excel sat 2020. The F value indicated equality of variances. It was calculated as: F = (Sum of squares between group/degree of freedom)/(Sum of squares within group/degree of freedom). The species richness (S) and the Shannon biodiversity index (H’) were used to evaluate the diversity of AMF in the studied soils through different inoculation treatments after 6 months of culture using (PC-Ord 5.0). The AMF biodiversity in studied soils must be close to H_max_ to reflect a high biodiversity.

## 5. Conclusions

The present investigation highlights the positive effectiveness of indigenous mycorrhizal inoculum on *T. articulata* plant growth and soil microbial biomass in arid and semi-arid saline soils. The efficiency of indigenous AMF inoculum over a commercial one as a bio-fertilization strategy in order to ensure the success of revegetation operation in degraded saline soils with *T. articulata* is demonstrated. The significant interactions between *T. articulata* and AMF indigenous species underline the importance of selecting the appropriate host-AMF combination for improving performance of revegetation worldwide under saline conditions. The current study opens the way towards the on-site development of AMF inoculum using indigenous strains to perform a sustainable revegetation and even agriculture in arid and semi-arid saline soils.

## Figures and Tables

**Table 1:** Effect of AMF treatments on total AMF colonization of *T. articulata* cultivated in the four studied soils.

Mycorrhizal Rate/Studied Soils	LG (1.1 dS·m^−1^)			HB (2.1 dS·m^−1^)			DU (3.1 dS·m^−1^)			BG (4.5 dS·m^−1^)		
	NI	CI	AI	NI	CI	AI	NI	CI	AI	NI	CI	AI
Total mycorrhizal rate (%)	15.4 ^c^	15.1 ^c^	21.9 ^a^	14.7 ^c^	10.9 ^c^	26.7 ^a^	12.2 ^c^	11.8 ^b^	29.1 ^a^	9.4 ^b^	15.4 ^b,c^	33.2 ^a^
Total arbuscules (%)	8.1 ^c’^	11.6 ^b’^	16.6 ^a’^	9.3 ^c’^	5.6 ^c’^	12.2 ^a’^	8.6 ^c’^	8.8 ^c’^	13.6 ^a’^	4.2 ^c’^	6.6 ^c’^	16.6 ^a’^
Total vesicles (%)	11.2 ^c,†^	14.8 ^b,†^	25.7 ^a,†^	13.2 ^c,†^	9.2 ^b,†^	16.6 ^a,†^	10.3 ^b,c,†^	10.6 ^c,†^	22.1 ^a,†^	8.3 ^c,†^	11.6 ^c,†^	28.3 ^a,†^

NI: non-inoculated. CI: commercial inoculum. AI: Indigenous inoculum. Data are represented as mean ± standard deviation. Means were obtained from five replicates (*n* = 5). Different letters with according symbols indicate significant differences between treatments for each group separately according to the Tukey HSD test (*p* < 0.05). Values within a line followed by the same letter are not significantly different at *p* < 0.05.

**Table 2:** Pearson’s correlation coefficient between mycorrhizal colonization rates, shoot dry weight and the different measured parameters under AMF inoculation treatments.

	NI Treatment		CI Treatment		AI Treatment	
	MCR	SDW	MCR	SDW	MCR	SDW
SDW	0.5 *	–	0.7 *	–	0.9 **	–
RDW	0.6 *	–	–0.2	0.9 ***	0.8 **	0.9 ***
Shoot Phosphorus	0.6 *	0.6 *	0.7 *	0.5 *	0.9 **	0.7 *
Soil phosphorus	–0.3	0.9 ***	0.3	0.7 **	–0.8 **	0.2
Shannon index	0.3	0.1	0.3	0.2	0.9 **	0.4 **
AMF species richness	0.5	0.3	−0.4	–0.5	0.9 **	0.2 **
NLFA C16:1ω5	0.6 *	0.4 *	−0.1	–0.5	0.7 *	0.5 *
PLFA C16:1ω5	–0.00	0.6	−0.00	0.5	0.6 *	0.2 *
NLFA/PLFA C16:1ω5	0.6 *	0.3 **	−0.1	−0.3	0.6 *	0.00 *
Ergosterol	0.4	0.6	0.0	0.3	0.6 *	0.2 *
Gram positive bacteria	0.5	0.0	0.2	0.1	0.5 *	0.2 *
Gram negative bacteria	0.4	0.3	0.00	0.1	0.6 *	0.2 **

MCR: Mycorrhizal colonization rate. SDW: Shoot dry weight. RDW: root dry weight.* Correlation is significant at the 0.05 level. ** Correlation is significant at the 0.01 level. *** Correlation is significant at the 0.0001 level. NI: non-inoculated. CI: commercial inoculum. AI: indigenous inoculum.

**Table 3:** One-way ANOVA for specific factors with three inoculation treatment levels and their interactions with studied parameters.

Sources of Variation/Parameters			Mycorrhizal Rate	Shoot Phosphorus	Total Dry Weight	NLFA	PLFA	Ergosterol	Gram +	Gram −	Soil Phosphorus	AMF Spore Biodiversity
Soil salinity effect		R²	–0.461	0.135	0.313	0.564	0.304	0.161	0.329	0.409	0.139	0.78
		F	2.381	0.434	1.267	3.595	1.212	0.532	1.361	1.926	0.447	27.668
		Pr > F	0.042 *	0.904	0.302	0.005 **	0.331	0.838	0.257	0.095 *	0.896	0.001 *
Intergroup Inoculum sources	NI	R²	0.580	0.53	0.567	0.521	0.478	0.53	0.43	0.12	0.78	0.618
		F	15.5	3.56	31.23	2.15	1.99	1.52	2.89	–2.26	3.87	23.5
		Pr > F	0.05 *	*p* < 0.02 *	0.05 *	0.02 *	0.05 *	0.05 *	0.02 *	0.45 ns	0.02 *	0.05 *
	IC	R²	0.406	0.135	0.479	0.46	0.347	0.19	0.32	0.24	0.24	0.56
			3.17	1.9	32.15	1.091	1.12	0.51	1.36	–1.8	2.5	21.03
		FPr > F	0.009 **	0.8	0.05 *	0.1	0.12	0.8	0.1	0.19	0.4	0.05 *
	AI	R²	0.593	0.624	0.618	0.65	0.74	0.65	0.67	0.62	0.798	0.65
		F	14.9	4.34	7.62	4.12	2.39	2.11	2.06	6.19	21.34	2.758
		Pr > F	0.001 **	*p* < 0.05 *	0.002 **	0.002 **	0.05 *	0.05 *	0.01 **	0.02*	0.05*	0.001 **
Contrast	NI vs. IC		*p* < 0.04 *	*p* < 0.02 *	*p* < 0.05 *	*p* < 0.01 **	*p* < 0.03 *	*p* < 0.05 *	*p* < 0.98	*p* < 0.32	*p* < 0.311	0.02 *
	NI vs. AI		*p* < 0.02 *	*p* < 0.3 ns	*p* < 0.04 *	*p* < 0.03 *	*p* < 0.02 *	*p* < 0.812	*p* < 0.9	*p* < 0.19	*p* < 0.261 ns	*p* < 0.03 *
	IC vs. AI		*p* < 0.05 *	*p* < 0.05 *	*p* < 0.02 *	*p* < 0.01 **	*p* < 0.04 *	*p* < 0.05 *	*p* < 0.5	*p* < 0.78 ns	*p* < 0.385 ns	*p* < 0.04 *

NLFA: Neutral lipid fatty acid, PLFA: phospholipid fatty acids, R^2^: Coefficient of regression, F: F Value, *p*: significance level. * *p* <: 0.05, ** *p* < 0.01. ns: non-significant. NI: non-inoculated, CI: commercial inoculum, AI: indigenous inoculum.

## Data Availability

We confirm that all data are original and provided in tables and figures within the article.

## Supplementary Materials

The following are available online at https://www.mdpi.com/article/10.3390/plants10122716/s1, Table S1: Diversity and abundance of AMF species in soils collected six months after inoculation of T. articulata, Table S2: Chemical analysis of the soil used in the present study. Figure S1: DCA ordination of AMF distribution according to AMF inoculation treatment in studied saline soils.
