# Supplementary material for: Inoculum Sources Modulate Mycorrhizal Inoculation Effect on Tamarix articulata Development and Its Associated Rhizosphere Microbiota
Source: Plants (Basel). 2021 Dec 10;10(12):2716. doi: 10.3390/plants10122716 (PMC8707033; doi:10.3390/plants10122716)
Supplement: Supplementary file 1 [file plants-10-02716-s001.zip › plants-1370345-supplementary.pdf]

Table S1 Diversity and abundance of AMF species in soils collected six months after inoculation of *T. articulata*

AMF species	LG			HB			DU			BG		
	NI	CI	AI	NI	CI	AI	NI	CI	AI	NI	CI	AI
Total AMF spore number/g	6	7	17	7	6	14	4	7	13	8	7	16
S	5	4	7	5	4	7	4	4	5	5	5	6
H’	1.48	1.28	1.52	1.48	1.24	1.76	1.20	1.04	1.49	1.25	1.08	1.28
H_{max}	1.94	1.38	1.60	1.60	1.60	1.79	1.38	1.38	1.60	1.79	1.60	1.60
Funnneliformis geosporum	180	270	430	270	190	510	140	250	660	310	250	650
Septoglomus constrictum	140	150	380	130	160	440	90	190	370	230	160	70
Funneliformis mossseae	80	110	320	100	110	330	90	100	130	310	150	560
Funneliformis coronatum	nd	80	250	nd	nd	50	Nd	80	nd	50	10	300
Funneliformis caledonium	nd	nd	70	nd	nd	40	Nd	nd	nd	nd	Nd	60
Rhizoglomus fasciculatum	130	nd	110	60	nd	nd	Nd	nd	nd	nd	Nd	nd
Oehlia diaphana	nd	nd	nd	nd	10	20	Nd	nd	nd	nd	10	nd
Gigaspora gigantea	30	nd	90	130	nd	40	Nd	nd	30	nd	Nd	nd
Paraglomus sp1	nd	nd	nd	nd	nd	nd	50	nd	60	10	Nd	30

NI: non inoculated, CI: commercial inoculum, AI: Indigenous inoculum, S: AMF species richness; H : Shannon biodiversity index. Means of AMF abundance are obtained from five replicates per treatment (n=5).

Table S2 Chemical analysis of the soil used in the present study.

Sites/Parameters	Laghouat (LG)	Hassi-Bahbah (HB)	Djelfa (DU)	Boughzoul (BG)
EC ($ds.m^{-1}$)	1.1	2.1	3.01	4.5
pH $_{water}$	8.00	7.78	7.45	7.48
Total Nitrogen ($mg.g^{-1}$)	0.20	0.23	0.23	0.42
Available phosphorus ($mg.Kg^{-1}$)	0.14	0.44	0.11	0.21
AMF infective propagules ($MPN.g^{-1}$ dry soil)	5.3	4.6	2.8	2.33
Texture	Silty fine-clay	Sandy	Silty-sandy	Silty-sandy

MPN: Most probable number, EC: Electrical conductivity evaluated by degree simens.meter^{-1}

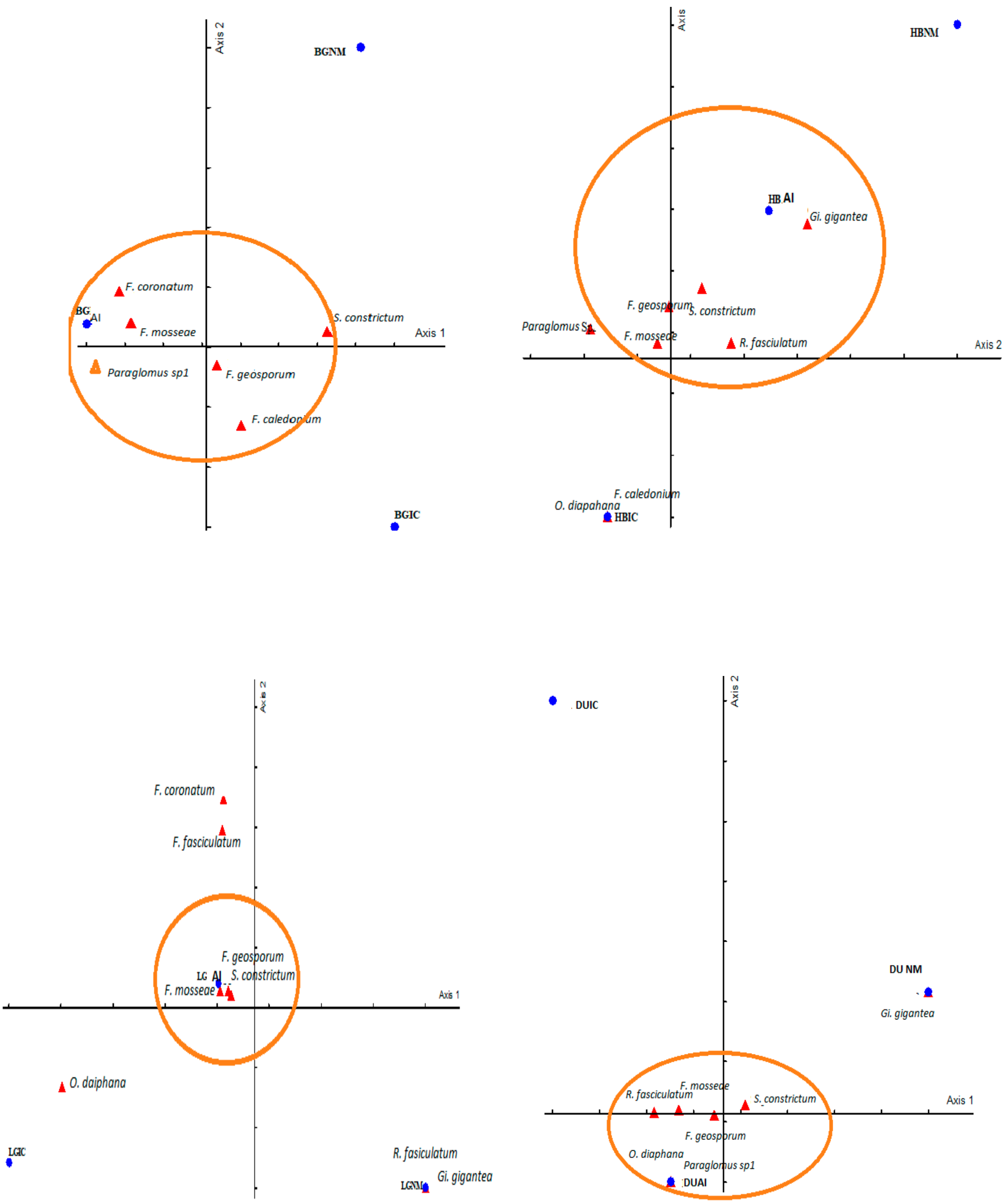

Figure S1: DCA ordination of AMF distribution according to AMF inoculation treatment in studied saline soils. Data of AMF was based on means of three replicates samples. Blue dots represent the AMF inoculas treatments. Read triangle indicate the identified AMF species. The proximity of read triangle to a treatment means that the corresponding AMF species is associated to the treatment. BG: saline site. BGNM: non-inoculated soil BG, BGIC: commercial inoculum in saline soil BG, BGAI: Autochthonous inoculum in saline soil BG. HBNM: no-inoculated soil HB, HBIC: Commercial inoculum in HB soil, HBAI: indigenous inoculum in HB. DUNM: Non-mycorrhizal DU soil, DUIC: commercial inoculum in DU, DUAI : indigenous inoculum in DU. LGNM : non-mycorrhizal soil LG, LGIC : commercial inoculum in LG soil, LGAI : indigenous inoculum in LG soil.
